# Anomalous Ataxies

**Published:** 1891-02-07

**Authors:** 


### ANOMALOUS ATAXIES.

The multiplicity of the conditions, comprised for convenience under the names of tabes dorsalis and locomotor ataxy, is well shown by two cases, described respectively by Drs. Goldscheider and Bäumler, in one of which there was no loss of sensation, while in the other only those movements were deranged which depended on sensation for their execution. Dr. Bäumler's patient, aged 44, had suffered when fifteen from a contusion of the spinal column, followed by paraplegia, which, however, gradually passed off. Twelve years ago he began to suffer from pain in the sacrum and motor disturbances. On examination all the reflexes were found to be increased, and sensibility, especially muscular, much lowered. Movements of the upper limbs demanding sensibility for their due performance were remarkably inco-ordinated, but in ordinary automatic movements no such loss of control was manifested. Goldscheider's patient had for two years suffered from numbness and paræsthesiæ; after a few months she could no longer stand with her eyes closed, and could scarcely mount the stairs. Romberg's and Westphal's signs were most pronounced, but ordinary methods of examination failed to detect any derangement of cutaneous sensibility or consciousness of passive movements, though special and more delicate tests did so to a slight degree.

